# Solvent selective gelation of cetyltrimethylammonium bromide: structure, phase evolution and thermal characteristics

**DOI:** 10.1098/rsos.231487

**Published:** 2024-04-03

**Authors:** Chapireddy Nagarjuna, Illa Ramakanth

**Affiliations:** ^1^ Department of Chemistry, School of Advanced Sciences, VIT-AP University, Amaravati, Andhra Pradesh 522 241, India

**Keywords:** gels, self-assembly, binary solvent mixtures, viscoelastic properties, thermal properties

## Abstract

We report herein the gelation behaviour of cetyltrimethylammonium bromide (CTAB), a cationic surfactant, in a variety of solvent compositions. A turbid gel of CTAB in a binary solvent mixture at a critical composition was observed to be 1 : 3 v/v toluene : water. The molecular structure of the as-formed gel was investigated by X-ray diffraction and microscopic techniques, namely, optical and polarizing microscopy, scanning electron microscopy and small-angle X-ray scattering (SAXS). The phase evolution has been studied using UV–visible transmittance measurements and the thermal characteristics of the gel by differential scanning calorimetry measurements. SAXS studies, in conjunction with molecular modelling, revealed the gel to assemble as lamellae with high interdigitation of bilayer assembly of CTAB molecules with predominant non-covalent interactions, where the gel lamellae were inferred from the interplanar spacings. Rheological studies revealed the viscoelastic nature of the CTAB gels. The ability to form a gel has been evaluated in several polar solvents, such as methanol and chloroform, and non-polar solvents, such as toluene and carbon tetrachloride.

## 1. Introduction

In the sense of the natural properties of molecules, the assembling nature and companionship among molecules are demonstrated by elucidating the correlation between the spatial arrangement and functioning of the atom in a molecule. Self-assembling of corpuscles to figure as gentle stuff enlarged attentiveness owing to the molecule’s wide variety and simple tunable nature [1–4]. Owing to the formation of various stacked arrangements of aromatic molecules, they can prefabricate into transcendent nanomaterials [3–6]. Numerous transecting agents can be synthesized because of the structure’s adaptability. Selectively, gels are the non-fluid colloidal systems that are competent to reserve a viscoelastic material in which an extensive portion is composed of liquid form. In general, gels are classified into hydrogels, organogels and aerogels based on the nature of the solvent. In the recent past, there has been a curiosity in organogelators from low-molecular-weight gelators (LMOGs), which produce such gels with numerous carbon-based solvents. LMOGs and their supramolecular gels have attracted much attention as to their possible applications in the fields of chemistry [5–12].

Considering various non-covalent interactions, the supramolecular configurations are pre-fabricated depending on the nature of the solvent. Usually, gels are synthesized by cooling the liquid phase of LMOGs beneath the formation of its dense nature involving intermolecular forces among the molecules [13–18]. The coagulation of solute forms hydrogels in the presence of water, and gelators are responsible for solidifying nature. The strong bonding between the gelator and solvent makes the solution ungelated and also averts condensation [1,13–16]. Hydrogels exhibit hydrophilicity, amphiphilicity, biodegradability, tissue adhesiveness, elasticity and stimuli responsiveness by modifying their permeable three-dimensional system, used in the equalization of nanoparticles and organic photochromatic molecules, light harvesting materials, catalysis, regenerative medicine, optoelectronic areas and environmental science [17–32].

In minute miscellaneous-grained phases, centrifugal forces form a three-dimensional network in swamped solutions and disable the solvent particles comprehensively. The dual combination of solvents toluene-n-heptane arbitrated by n-lauroyl-L-alanine has been reported [1]. Organogel hydroxyoctadecanoic acid was reported by adding selective organic solvents [4]. L-glutamic acid-based organogelator was developed by the addition of amides to Boc-L-glutamic acid with octadecylamine [8]. In the sol-gel copper (II) acetate hydrate [Cu(COOCH_3_)_2_.xH_2_O, 98%] with monoethanolamine (MEA), a stabilizer was prepared, and the precursor was dissolved in a mixture of isopropyl alcohol and 2-methoxy ethanol in several ratios [33]. The binary solvent gel is observed by combining cyclodextrins with drugs such as doxycycline, and pyrene was characterized as a drug delivery carrier [34]. Mostly, the solvents, water, methanol, isopropanol, chloroform, etc. are used to form organogels or xerogels. The addition of an enteroceptor to the gelation mixture exhibits positive responses to the applied extrinsic factors used in the areas of sensing [1], drug industry [12], biomaterials [14], material sciences [5], nanochemistry [20], oil recovery [28], polymer chemistry [30], etc.

The charge transfer active gel has been prepared and reported from the combination of cationic cetylpyridinium chloride with bola-amphiphile 6-aminocaproic acid. The objective of the present work is to investigate the self-assembly behaviour of cetyltrimethylammonium bromide (CTAB) and its gelation ability in various solvent combinations. Following our recent work [35] on pH-sensitive smart gels formed from CTAB, the present investigation explores the physico-chemical, structural and thermal properties of the CTAB gels from binary solvent mixture of toluene and water. The as-prepared CTAB gel has been organized into a lamellar gel owing to the presence of hydrophobic interactions in the proposed fibrous gel network structure [36].

## 2. Material and methods

**Scheme 1.**
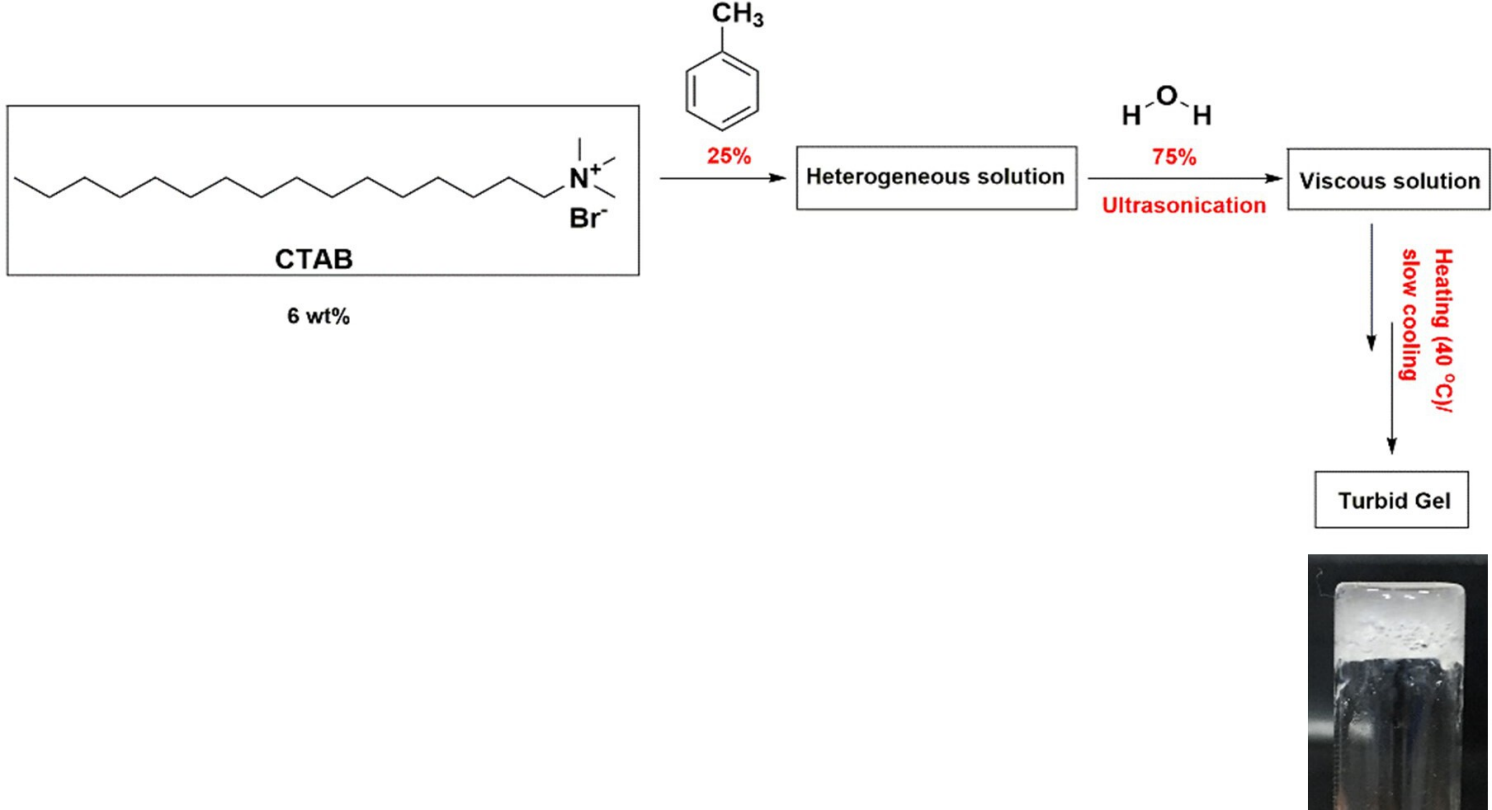
Methodology for CTAB gelation in 25 : 75 v/v toluene : water solvent composition.

CTAB 99% and toluene 99.5% were purchased from SRL Pvt. Ltd, India. Toluene acts as a structure-directing agent through the balance of weak intermolecular forces in interactions. Apart from that, toluene is a non-polar solvent, whereas water is polar. When both toluene and water are mixed together, the mixture becomes immiscible. When toluene is added to CTAB, it forms a milky emulsion followed by turbid gel formation upon the gradual addition of water. A digital conductivity meter (Mettler Toledo FiveEasy Plus FP30, Switzerland) with a cell constant of 1 cm^−1^ was used to perform conductivity measurements. Ultrapure deionized water (LAB-Q, India) was used in preparing the aqueous solutions at 25 ± 0.1°C. The Shimadzu (Japan) UV-1900 double-beam spectrophotometer was used for recording electronic absorption spectra with a Bexco UV quartz cuvette of 10 mm path length. The procedure for gelation has been depicted in Scheme 1.

Small-angle X-ray scattering (SAXS) of gel samples was performed with a Bruker-AXS NanoSTAR (Germany) instrument using CuKα emission, estimated at 45 kV/100 mA with two-dimensional gas detection (HI-STAR). SAXS analysis in the ‘q’ range of 0.05–0.72 Å^−1^ for the 2θ range between 0.05° and 12.5°.

X-ray diffraction (XRD) measurements were carried out using an Empyrean (UK) instrument using CuKα, carried at 50 kV/40 mA, and measured in the 2θ range between 5° and 120°.

Scanning electron microscopy pictures were captured on a Zeiss scanning electron microscopy (Germany) by treating the sample with carbon using a vacuum sputter-coater, followed by desiccation at 25°C in the radiation phase under a lesser vacuum. The range at which the ray of light is 0.02–20 kV is used to figure out the sample.

Optical microscopy images were acquired on a Nikon microscope (Japan) equipped with brightfield and darkfield observation CFI TU PLAN FLUOR optics and an Eclipse LV150N model.

Differential scanning calorimetry (DSC), Perkin Elmer (USA), experiments were carried out for CTAB gel at pH 7, which resembles its property of reversible nature to heat. From −70 to 100°C (first heating), 100 to −70°C (cooling) and then −70 to 100°C (second heating) were the respective cycles taken for CTAB in toluene : water.

Rheology: The multi-phase properties of the gelation were analysed using an Anton Paar 100 (Austria) rheometer having plane algebra (CP 25-2* with a 2° cone angle) at 25°C with a vibratory occurrence of 1 Hz at a magnitude of pressure of 50 Pa.

## 3. Results and discussion

**Figure 1.**
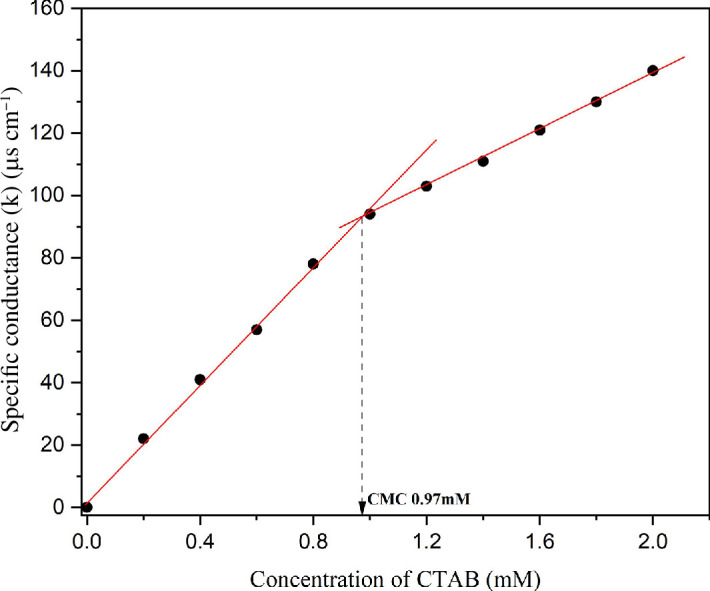
Conductivity profile depicting 0.97 mM CMC for CTAB at 25°C.

The critical micelle concentration (CMC) value of 0.97 mM was measured using conductivity measurements; water shows a conductance of 0.05 microsiemens (µS) which is consistent with the literature-reported value of 1.0 mM [37]. CTAB forms a reverse micellar phase in low-dielectric solvents, namely, toluene and chloroform, in the presence of water (figure 1) at CMC at 25°C [38].

In the present investigation, self-assembled aggregates were formed by adding a small quantity of water to a solution of reverse micelles; a turbid phase was evolved at an optimized H_2_O proportion of 750 µl, equivalent to a 6 wt% CTAB gel concentration in 1 : 3 v/v toluene : water (cf. electronic supplementary material, table S1). The successive addition of water (710–760 µl) resulted in a significant rise in solution viscosity, followed by the development of a gel phase and a significant decrease in transmittance value from UV–visible spectroscopic measurements. Water was gradually added to the CTAB–toluene solution, which was then sonicated and cooled slowly to allow a viscous gel to develop.

**Figure 2.**
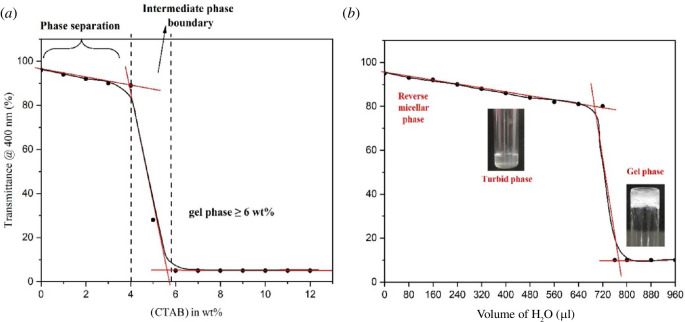
(*a*) UV–visible measurements in transmittance mode as a function of CTAB concentration indicate the formation of CTAB gels (critical concentration greater than or equal to 6 wt%). (*b*) Transmittance measurements indicating CTAB gel-phase evolution after the addition of the required volume of water.

**Figure 3.**
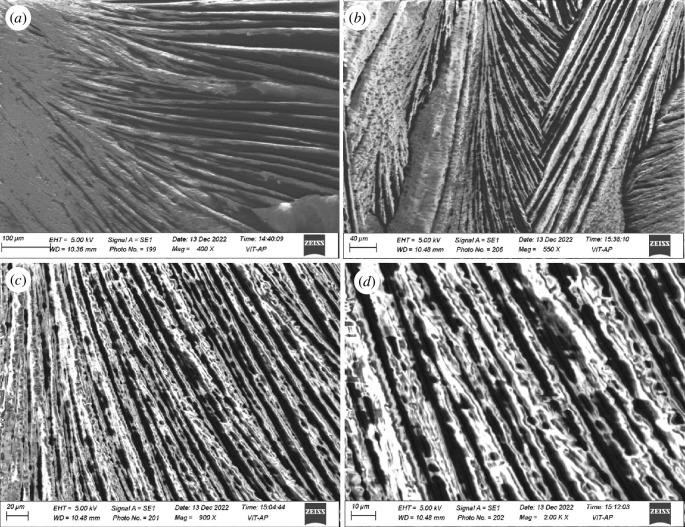
(*a*–*d*) SEM images of CTAB gel show aligned fibrous bundles with 10–60 µm width.

**Figure 4.**
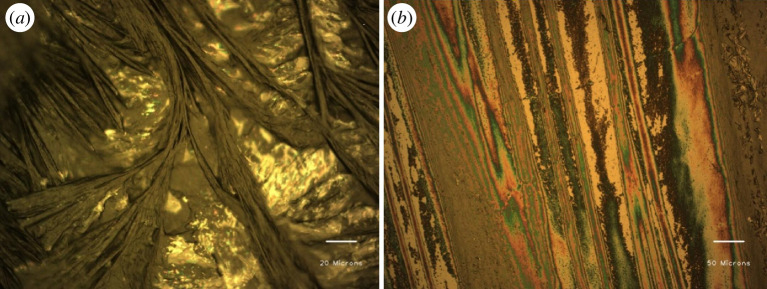
(*a*) CTAB gel phase under polarizer mode (×20). (*b*) Optical microscopic picture from the xerogel exhibiting birefringence with a polarizer (×50) (6 wt% CTAB in toluene : water: 1 : 3 v/v).

The results from UV–visible transmittance measurements are shown in figure 2*a*. The transition from a dense gel phase into a colloidal solution was observed with successive addition of water beyond 960 µl. The sample glass vials were firmly sealed in order to prevent the evaporation of the toluene solvent. The CTAB in a binary solvent system developed a transparent solution that was not viscous and transformed into a viscous gel after ultrasonication and slow cooling. The as-formed gels have low optical transparency and are thus confirmed through the vial inversion test (cf. figure 2*b*). Scanning electron microscopy (SEM) images of CTAB gels indicated aligned fibrous bundle networks formed with a high ratio of their length to their width, i.e. 10–60 μm in width and several microns in length, as depicted in figure 3*a*–*d*. The xerogels showed significant birefringence under polarized radiation, as illustrated in figure 4*b*.

**Figure 5.**
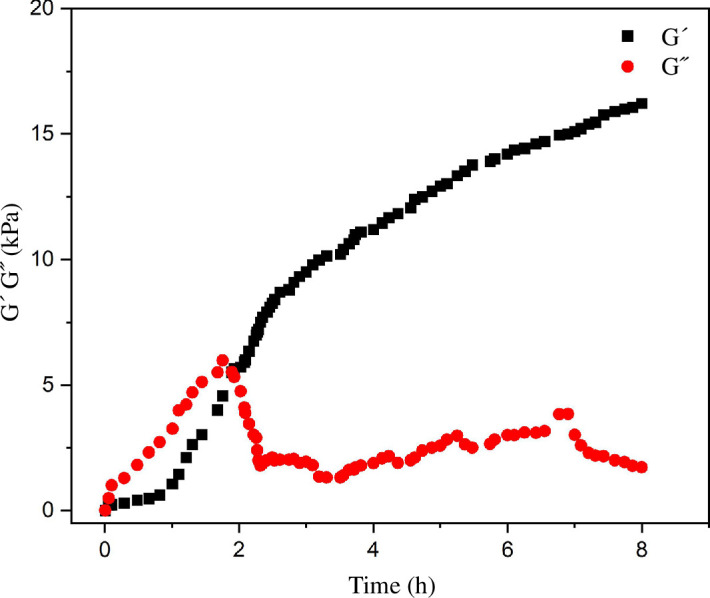
Rheology profiles showing variation of *G*′, *G*″ with gel formation time for CTAB gel in toluene : water (1 : 3 v/v). Stress amplitude (σo) of 50 Pa was used.

Rheology measurements were performed to investigate the viscoelastic characteristics of gels consisting of CTAB in a binary solvent mixture of toluene : water. Since these molecular gels have a viscoelastic nature, they store and release energy, as measured by the storage modulus *G*′ and loss modulus *G*″, respectively. Figure 5 depicts the rheology results acquired for CTAB in toluene and water (1 : 3 v/v). The preliminary results of *G*′ and *G*″ indicated that the system would behave like a viscous liquid. When the period of gelation is increased (duration greater than 1 h), a sudden increase in G′, considerably more significant when compared with *G*″, suggests the development of a fibrous aligned bundle structure. After a few hours in the plateau zone, both *G*′ and *G*″ of modulus remained steady and rose with time.

**Figure 6.**
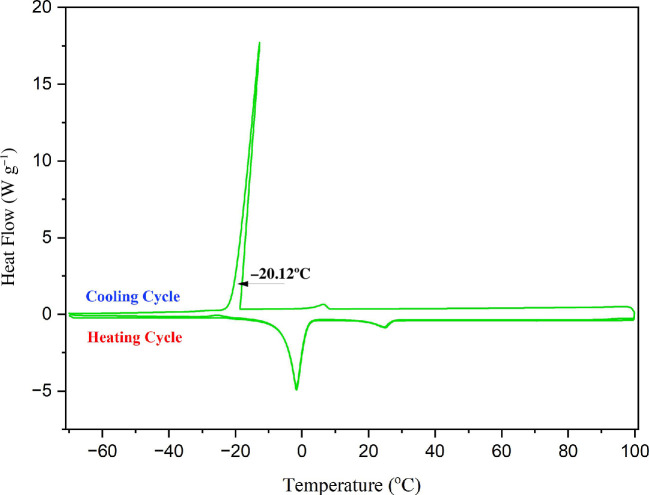
DSC thermogram of the CTAB gel from toluene : water (6 wt% CTAB in toluene : water (1 : 3 v/v)). Respective scans were performed starting from −70 to 100°C (first heating), 100 to −70°C (cooling) and then −70 to 100°C (second heating) in the case of CTAB in toluene and water.

Thermo-reversible gel–sol transitions have been studied using DSC. Figure 6 depicts the exothermic peak, which develops heat by decreasing its temperature in the cooling DSC curve, as well as the endothermic peak, which demonstrates a heat-absorbing nature by increasing its temperature in the heating DSC curve of the CTAB gel. At 6 wt% CTAB in toluene and water (1 : 3 v/v), the peaks at which molecules get fused and precipitated from the heating and cooling ranges for the gelation of CTAB are evidenced from DSC micrographs. The intense cooling curve, in the presence of universal solvent water, is the immense projection produced owing to the formation of frost-like particles, and the hump is reflected at −20.12°C, as shown in figure 6.

**Figure 7.**
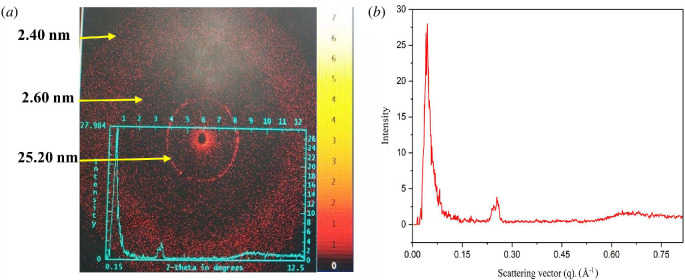
(*a*) SAXS pattern and (*b*) spectrum of CTAB gel formation at 25°C. The figure represents the d-spacings calculated using d = λ/2sin θ.

Using scattered light from the CTAB samples, SAXS has been widely used to study the internal organization of the gels at the molecular level and the packing in CTAB gel. The attractive and repulsive forces within the molecules integrated with the binding effects of the gel can be taken advantage of by the molecular arrangement in the structure of the dense-natured fibres [39]. The molecular composition of gels consisting of organic liquid phase and water has been illuminated by wide-angle X-ray powder diffraction in a highly competent manner [40,41]. The filling up of the gel molecules either in stretched or hooked configurations or by the available interlinking of a group of substituents attached can be regulated by differing interplanar spacing ‘d’ that extends to the size of the molecule, which corresponds to the extensive intervals in the complex having a translucent nature, by considering the powder XRD sequence. David *et al*. explained that the distances of the ensuing SAXS peaks were used to describe the lamellar (multi-phase colloidal structure) packing (1, 1/2, 1/3) from the hexagonal (1, 1/√2, 1/√3) filling of the molecules [42]. In order to investigate the microstructure of CTAB gel in toluene and water (1 : 3 v/v), a SAXS technique is used. SAXS plots and periodic diffraction peaks from figure 7 show that the estimated interplanar spacings, maintaining a ratio of 1 : 1/2 : 1/3, exhibit lamellar structure for the gels [41]. The interplanar spacings derived from small-angle scattering peaks and scattering vectors are shown in tables 1 and 2. Similar work on organogels from primary alkyl amines as gelators was reported by George and Weiss [[43]]. The molecular organization in a gel was established by correlating the interplanar spacing (d) values with the lengths of the molecules, which was supported by molecular modelling in the subsequent section [44].

**Table 1.** Interplanar spacings (d) calculated from the small-angle peaks in SAXS of CTAB gelation.

scattering angle (2θ/°)	interplanar spacing, d = λ /(2 sinθ) (nm)
0.20	45.30
0.35	25.20
0.56	15.70
0.62	14.21
1.16	7.60
3.39	2.60
3.51	2.51
3.58	2.40

**Table 2.** Interplanar spacings (d) calculated from the scattering vectors in SAXS of CTAB gelation.

scattering vector (q), Å^−1^	intensity (*I*)	interplanar spacing, d = 2 π /q (nm)
0.0140	0.65	44.85
0.0250	1.55	25.12
0.0400	26.72	15.70
0.0442	27.98	14.20
0.0817	4.97	7.60
0.2396	2.12	2.60
0.2500	2.70	2.51
0.2601	3.79	2.40

### 3.1. Modelling of the cetyltrimethylammonium bromide gelation

**Figure 8. F8:**
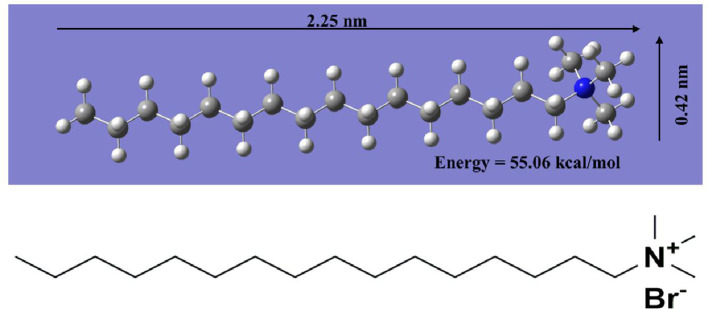
CTAB molecule model after energy minimization from density functional theory energy optimization using B3LYP/6-31G++. The respective chemical structure is shown below.

[Fig F8] Figure 8 depicts the geometrically optimized CTAB structure.

**Figure 9.**
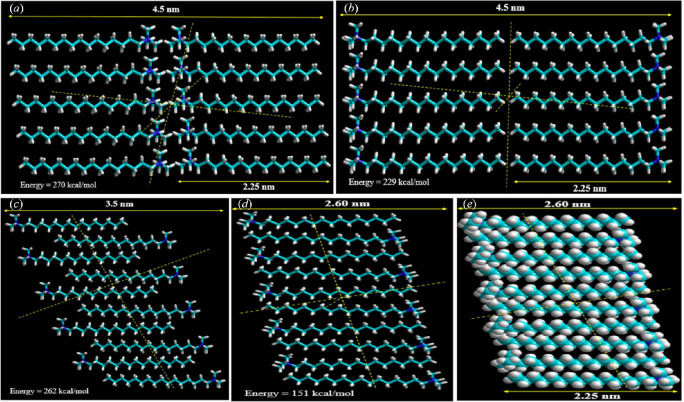
Bilayer packing model at the molecular level in CTAB gelation with MM+ optimization and energy-optimized assembly structures depicting (*a*) head-to-head, (*b*) tail-to-tail, (*c*) loosely packed, (*d*) the bilayers with high interdigitation and (*e*) the Corey–Pauling–Koltun (CPK) organization of high interdigitation of the CTAB bilayer assembly.

The SAXS investigations revealed four probable molecular packings developing a bilayer assembly with periods 4.5, 4.5, 3.5 and 2.6 nm, viz. (*a*) head-to-head, (*b*) tail-to-tail, (*c*) loosely packed and (*d*) the bilayers with high interdigitation, as depicted in figure 9. The MM+ force field was used to optimize the constructed bilayer structures with the energy-minimized structure of the CTAB molecule [45]. The spacing across alkyl tails had been fixed to be 0.45 nm for all the *trans* configurations in the organized molecular models. As a result, the molecular configuration with a minimized total energy equivalent to 151 kcal mol^−1^ indicated a strong interdigitation of the bilayers (figure 9*d*). The interplanar spacing was measured to be 2.6 nm, which is less than twice the expanded length of a CTAB molecule but greater than the length of a single CTAB molecule. As a result, the gel lamellae are composed of densely interdigitated bilayer structures of CTAB molecules linked together in a network with significant hydrophobic interactions.

**Figure 10.**
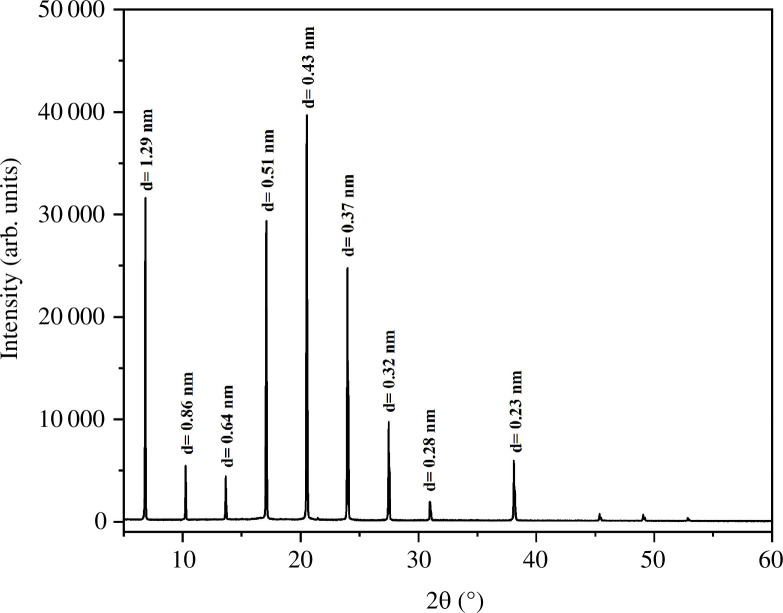
XRD profile of the gelation from CTAB.

Furthermore, the broad angle area of the CTAB gel XRD peaks (figure 10) revealed a succession of reflection peaks, supporting the hypothesis that long alkyl chains generated highly ordered layer packing by protracted interdigitation through hydrophobic contact, as evidenced by the Corey–Pauling–Koltun (CPK) organized assembly in figure 9. Figure 9 shows the energy-minimized ball-and-stick models of various bilayered packing arrangements of CTAB molecules. SAXS measurements indicated that CTAB gels had conceived a three-dimensional network with a high interdigitation of the CTAB bilayer assembly.

## 4. Conclusions

CTAB gelation from toluene : water, chloroform : water, dichloromethane : water binary solvent combinations at a specific 1 : 3 v/v composition ratio was observed. At a specific binary solvent composition, while a turbid gel from toluene was observed, chloroform and dichloromethane at pH 7 resulted in a very weak viscous gel, respectively. A CTAB gel network eventually formed as a result of phase transformation from a reverse micellar phase to a turbid viscous phase caused by varying the solvent composition. The structural characteristics of the aforementioned system were studied using molecular modelling, UV–visible transmittance, SAXS and SEM techniques. The gels maintained a three-dimensional network with a high interdigitation of bilayer arrangement of CTAB molecules having a 2.6 nm thickness, as shown by molecular modelling in combination with SAXS experiments. A lamellar organization of the gel was revealed by the periodic diffraction peaks from the SAXS analysis. These supramolecular could be used as templates for assembling nanoparticles and quantum dots, in tissue technology, biomedicine and biosensors.

## Data Availability

Data deposited at Dryad [46]. Supplementary material is available online [47].
